# Deep learning for blood glucose level prediction: How well do models generalize across different data sets?

**DOI:** 10.1371/journal.pone.0310801

**Published:** 2024-09-25

**Authors:** Sarala Ghimire, Turgay Celik, Martin Gerdes, Christian W. Omlin

**Affiliations:** 1 Department of Information and Communication Technologies, Centre for e-Health, University of Agder, Grimstad, Norway; 2 Department of Information and Communication Technologies, Centre for Artificial Intelligence Research (CAIR), University of Agder, Grimstad, Norway; Jhargram Raj College, INDIA

## Abstract

Deep learning-based models for predicting blood glucose levels in diabetic patients can facilitate proactive measures to prevent critical events and are essential for closed-loop control therapy systems. However, selecting appropriate models from the literature may not always yield conclusive results, as the choice could be influenced by biases or misleading evaluations stemming from different methodologies, datasets, and preprocessing techniques. This study aims to compare and comprehensively analyze the performance of various deep learning models across diverse datasets to assess their applicability and generalizability across a broader spectrum of scenarios. Commonly used deep learning models for blood glucose level forecasting, such as feed-forward neural network, convolutional neural network, long short-term memory network (LSTM), temporal convolutional neural network, and self-attention network (SAN), are considered in this study. To evaluate the generalization capabilities of each model, four datasets of varying sizes, encompassing samples from different age groups and conditions, are utilized. Performance metrics include Root Mean Square Error (RMSE), Mean Absolute Difference (MAD), and Coefficient of Determination (CoD) for analytical asssessment, Clarke Error Grid (CEG) for clinical assessments, Kolmogorov-Smirnov (KS) test for statistical analysis, and generalization ability evaluations to obtain both coarse and granular insights. The experimental findings indicate that the LSTM model demonstrates superior performance with the lowest root mean square error and highest generalization capability among all other models, closely followed by SAN. The ability of LSTM and SAN to capture long-term dependencies in blood glucose data and their correlations with various influencing factors and events contribute to their enhanced performance. Despite the lower predictive performance, the FFN was able to capture patterns and trends in the data, suggesting its applicability in forecasting future direction. Moreover, this study helps in identifying the optimal model based on specific objectives, whether prioritizing generalization or accuracy.

## Introduction

Diabetes is a chronic disease that requires high care and attention to keep blood glucose level (BGL) within a safe range. Due to the influence of several factors such as meal intake, physical activities, insulin, stress, or illness, controlling BGLs is challenging [1]. Thus, self-care, compliance with recommended lifestyle, and timely blood glucose (BG) measurement play a vital role [2]. To measure and regulate BGLs and avoid short or long-term complications, a continuous glucose monitoring (CGM) system has been widely adopted in recent years [3]. CGM measures BGLs in the interstitial fluid under the skin and estimates the plasma glucose with a higher sampling rate, producing a considerable volume of data that could be used in different data-driven models to infer future values for early prognosis and prevention of complications [4]. BGL prediction is a critical aspect of diabetes management. Accurate and timely predictions make it possible to take actionable initiatives to reduce the adverse effects of hyper or hypo-glycemic events and optimize the decisions regarding diet, exercise, and treatment plans [5].

With the advancement in CGM, several physiological and data-driven models for BGL prediction have emerged as promising tools to provide real-time forecasts [2–6]. While physiological models can mathematically describe BG kinetics and metabolism [3], their complex architecture requires knowledge about an individual’s physiological mechanisms [7]. Several physiological parameters are estimated and adjusted in advance through an exhaustive search, relying on the limited observed data, which are error-prone and time-consuming [6]. On the other hand, the data-driven approaches depend solely on the self-monitored historical data and require less knowledge about physiological metabolism. Thus, these approaches have been attracted as complementary to the traditional physiological model [8].

Considerable research has been done using data-driven models for the development of BGL prediction algorithms [7, 9]. However, most of the predictive models were assessed using diverse datasets available publicly or proprietary datasets with different input variables and time horizons, making it difficult to compare, analyze, and discover the best-performing models. Also, most of the studies utilized datasets with few subjects and data collected over a short duration [9]. These datasets lack wide variations of glucose dynamics that could have been captured either by distinct subject categories with ample size or the samples acquired over a sufficiently long monitoring period. Owing to this fact, existing works could not verify their generalization capabilities, nor was the generalization capability examined [10–15].

Very few studies contributed to comparing the performance of state-of-the-art methods experimentally [8, 16, 17]; instead, systematic reviews have been conducted, where methods are reviewed rather than doing the implementation [6, 7, 9, 18]. The experimental comparisons in [8, 16, 17] rely either on very few sample populations or data collected over a short duration. In addition, the analysis was solely with a single dataset, where no models were assessed for generalizability, the most crucial factor to consider for realizing real-world scenarios or use in universally across different clinical settings.

**Fig 1:**
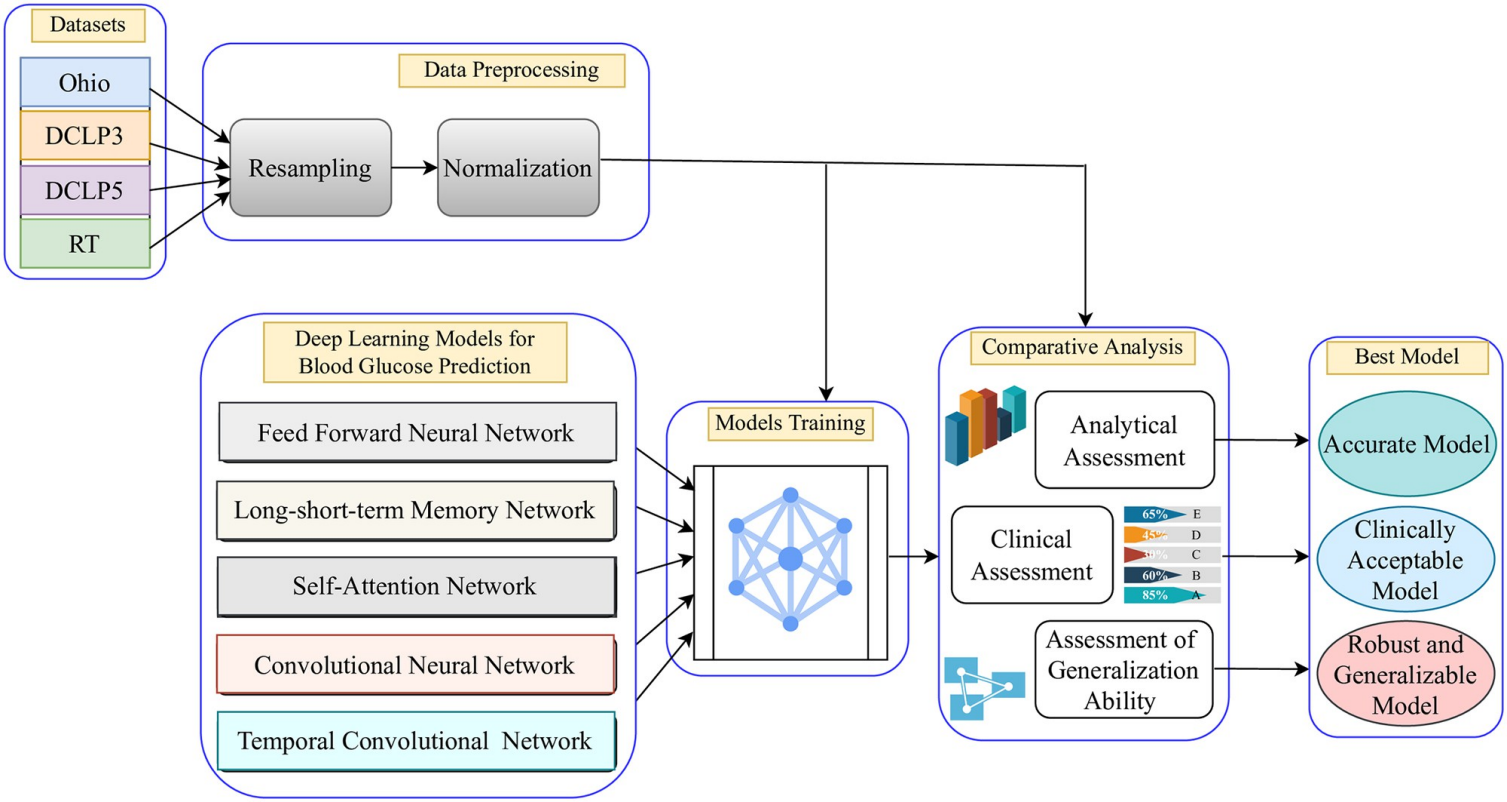
A schematic diagram of the proposed study representing overall workflow.

To address the aforementioned issues, we performed a comprehensive analysis of various deep learning models in forecasting BGLs using various open datasets as shown in the workflow diagram in Fig 1. The datasets encompass diverse features like samples from different age groups, with or without automated therapy, distinct sample size, and sample collection duration contributing to wide-ranging BG dynamics, a crucial factor in training a model to create a robust and versatile model in the practical context of BGL prediction. As shown in Fig 1, the deep learning models extensively employed in BGL prediction [7] and for time series problems [19] are considered for the comparison. The models are trained solely on historical BG data of the most commonly used datasets, OhioT1DM [20], RT [21], DCLP5 [22], and DCLP3 [23], with different prediction horizons (PHs) and evaluated based on the prediction accuracy. The datasets are preprocessed before using for training. The prediction accuracy is assessed using standard benchmarked performance metrics in BGL prediction models. Further, quantitative bias analysis is carried out to assess model performance and fairness across different datasets. The performance is evaluated over a PH of 30-minute and 60-minute, a commonly employed time frame for BGL predictions [1]. This time horizon enables timely intervention to avoid unwanted glycemic events [11]. The finally evaluated models are then considered accurate or clinically acceptable or the robust and generalizable model as shown in Fig 1.

To the best of our knowledge, this is the first comparative study that compares different deep learning models using diverse datasets of real patient data of type I diabetes, including children, adolescents, and elderly populations, for predicting BGLs and assessing their applicability and generalizability across diverse contexts. The primary aim of this study is to evaluate the performance of models across various patient groups, ensuring their reliability in real-world diabetes care, which involves a diverse population of diabetes patients with varying conditions and blood glucose dynamics. Thus, rather than focusing on developing or deploying a new optimized model, our emphasis is on evaluating established models used for predicting blood glucose levels and exploring their capacity to generalize across different unseen datasets. The novelty of this study lies in its comparative analysis, providing insights into how well these models apply to a diverse range of diabetic patients. Thus, the question that this study seeks to answer is: How well do different deep learning models generalize across diverse datasets and demographics for blood glucose prediction? We anticipate that the findings presented in this study will help identify the model that performs best across diverse datasets, especially in healthcare setting for different patients groups. It offers empirical insights for researchers to know how different models behave and how they can be applied in different circumstances. It helps to opt for the best model based on the priority of one’s specific goal, whether on generalization, robustness, or accuracy.

## Related works

This section briefly discusses the deep learning networks that are most significantly used in the current literature as well as in similar time series predictions [19, 24, 25]. We chose to focus exclusively on deep learning networks to explore the latest advancements in this field and assess their capability and effectiveness. We believe this study can provide insights that will help enhance the robustness of these models. The research conducted so far on these models are summarized in Table 1.

**Table 1:** Summary of the state-of-the-art BGL predictive methods.

Models	Datasets (No. samples, sample duration), Input features	Outperform	RMSE (PH: 60min)	Description
**FFN** [27]	T1DM (n = 12, 7 days), CGM	SVR,RF,DT	15.33	BGL data with time-domain attributes used as input to the FFN outperformed all other models.
**FFN** [28]	T1DM (n = 12, 14 days), CGM	SVR,AR,ELM	9.03	Using previous values along with their predictions as input to the FNN outperformed all other models.
**FFN** [29]	T1DM (n = 27, NA), CGM, Insulin, emotions, nutritions, activities	NA	43.9 (for PH: 75 min)	Real time prediction of BGL in patients with Type 1 diabetes for PH of 75min.
**LSTM** [3]	RT (n = 451,26 weeks), CGM	FNN, RNN, Autoregressive	32.38	Explored large dataset with heterogenous cohort of patients. LSTM outperformed other models in both long and short term predictions.
**LSTM** [8]	Ohio (n = 12, 8 weeks), CGM,Insulin, Carb, Heart rate	RF,TCN	19.58 (for PH: 30 min)	Stable performance was seen with LSTM in direct method than in the recursive method.
**LSTM** [12]	Ohio (n = 12, 8 weeks), CGM, Insulin, Carbs, Fingerstick BGL	NA	30.89	Leveraged multitask learning for personalized BGL prediction, with improved result.
**LSTM** [15]	Ohio (n = 12, 8 weeks), CGM	NA	30.89	BGL prediction with ensemble learning approach using vanilla and bidirectional LSTM as base learners.
**SAN** [10]	Ohio (n = 12, 8 weeks), CGM	NA	31.52	Explored attention-based deep network and enhances personalized prediction by transferring knowledge from population to individual.
**SAN** [11]	ARISES (n = 12,6 weeks), ABC4D (n = 25, 6 months), Ohio (n = 12, 8 weeks), CGM	ARIMA,Bi-LSTM, SVR	Ohio: 31.07, ARISES: 35.40, ABC4D: 34.03	Explored attention-based recurrent network for personalized BGL predictions with model confidence via an evidential layer.
**SAN** [13]	ARISES (n = 12,6 weeks), ABC4D (n = 25, 6 months), Ohio (n = 12, 8 weeks), CGM	ARIMA,Bi-LSTM, SVR	Ohio: 32.54, ARISES: 35.55, ABC4D: 33.88	Explored attention based evidential RNN network to design IoMT-enabled wearable device in edge computing.
**CNN** [33]	T1DM (n = 733), T2DM (n = 147), GDM (n = 20, 3 days), CGM	Scratch CNN, RF	T1DM: 28.1, T2DM: 27.7, GDM: 29.7	Demonstrated that the fine-tuning method improved the predictive performance.
**CNN** [5]	T2DM (n = 40), Ohio (n = 12, 8 weeks), CGM	SAN,RNN	T2DM: NA, T1DM: 33.80	Explored transfer learning to tackle small datasets and used TimeGAN for data augmentation to address imbalanced data in BG event prediction. CNN outperformed SAN and RNN.
**TCN** [8]	Ohio (n = 12, 8 weeks), CGM,Insulin, Carb, Heart rate	RF, SVRLinear	19.97 (for PH: 30 min)	Compared the performance of several machine learning and classic autoregression with exogenous input models. TCN showed greater robustness to spurious oscillations in BG trajectories.

### Feed-forward neural network

**Fig 2:**
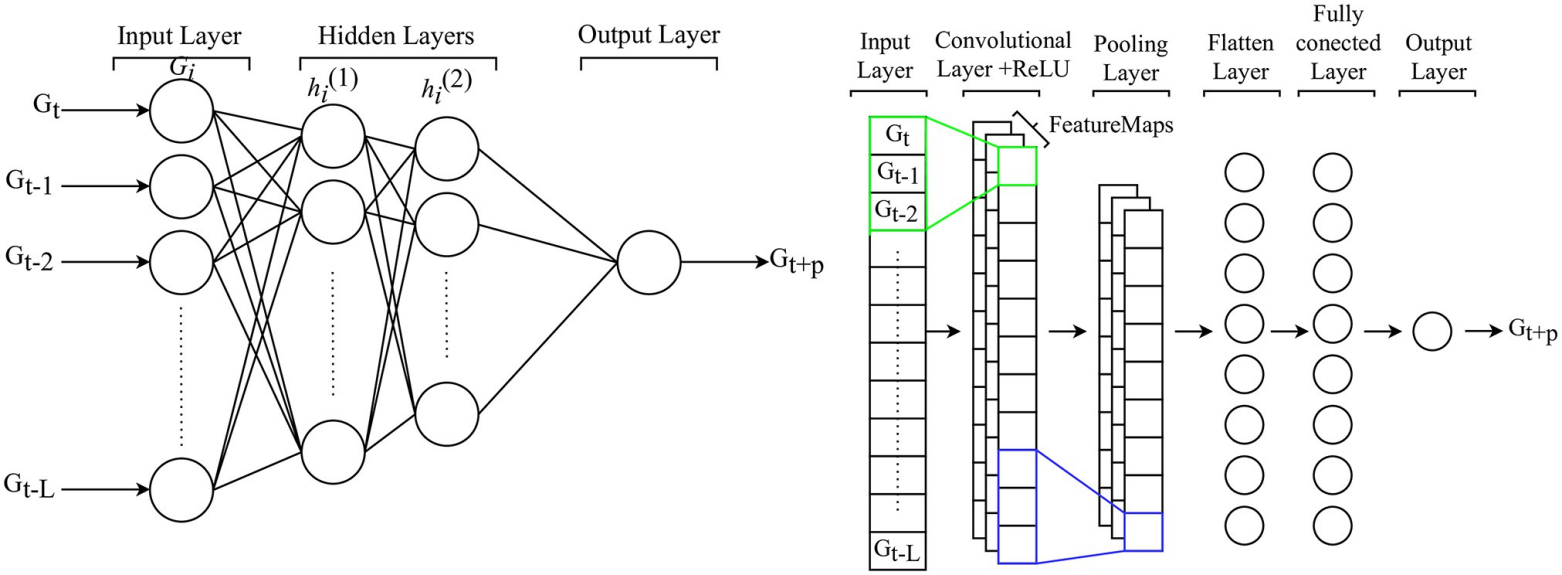
The schematic diagram of (Left) FNN which consists of an input layer, two hidden layers, and an output layer where data is propagated (feed-forward propagation) in one direction. (Right) CNN, composed of 1-D convolutional and pooling layers, connected with a fully connected layer for BGL prediction.

A feed-forward neural network (FNN) is a versatile and powerful deep learning model that processes data sequentially in one direction from the input to the output layer, offering solutions to diverse problems ranging from image recognition to time series forecasting [9]. For time series prediction, the FNN architecture analyzes sequential data over time, predicting future values or patterns without integrating feedback loops, distinctive from more complex recurrent neural networks (RNNs) [19]. It comprises an input layer receiving input data, one or multiple hidden layers with interconnected neurons, and an output layer generating outputs [26], as illustrated in the left side of Fig 2.

During training, FNN iteratively refines its weights and biases using optimization techniques to minimize the discrepancy between predicted and actual ground truth values [19]. Although FNNs cannot capture long-range dependencies compared to RNNs or TCNs, its simple network can also achieve good performance if the input variables are carefully selected and the preprocessing stage is given importance [19, 27–29]. Previous research demonstrated higher accuracy when CGM was solely utilized as an input for predicting BGL in an FNN-based model [27, 28] as shown in Table 1. Although the employed methods [27, 28] demonstrated improved performance in predicting blood glucose levels, the relatively small dataset size may restrict the generalizability of the derived conclusions. Furthermore, the methods’ dependence on manual feature extraction introduces a potential bottleneck, making their performance sensitive to feature quality and statistical analysis.

### Convolution neural network

Primarily identified for its ability in image processing [30], the convolutional neural network (CNN) has also been found applicable in analyzing time series data [26, 31]. In the context of time series analysis, a 1D CNN is designed to learn localized features or patterns embedded in the sequential data [32]. Leveraging convolutional layers embedded with filters, the network extracts hierarchical features, capturing immediate and prolonged patterns. Additionally, pooling layers help in dimensionality reduction, illuminating the salient features [30] The schematic diagram of CNN is shown in the right side of Fig 2.

Even though CNNs have not been extensively used for time series forecasting or BGL prediction, there are instances where they have been used, either independently in some studies [5, 33] or for feature extractions in conjunction with other models [1, 19]. These applications have demonstrated CNN to be suitable for BGL prediction or any other time series forecasting. Also, CNNs are designed for voluminous data and demonstrated accurate prediction when employed on larger datasets; because this study encompasses datasets of varying sizes, ranging from small to large, we anticipate CNNs offer a robust framework for assessing their performance across diverse data volumes, facilitating efficient data processing and salient feature extraction. Even though the studies [5, 33] utilized datasets from type 1, type 2, and gestational diabetic patients with large data sizes, demonstrating good results, none applied cross-dataset validation to assess the robustness of the models.

### Temporal convolutional network

**Fig 3:**
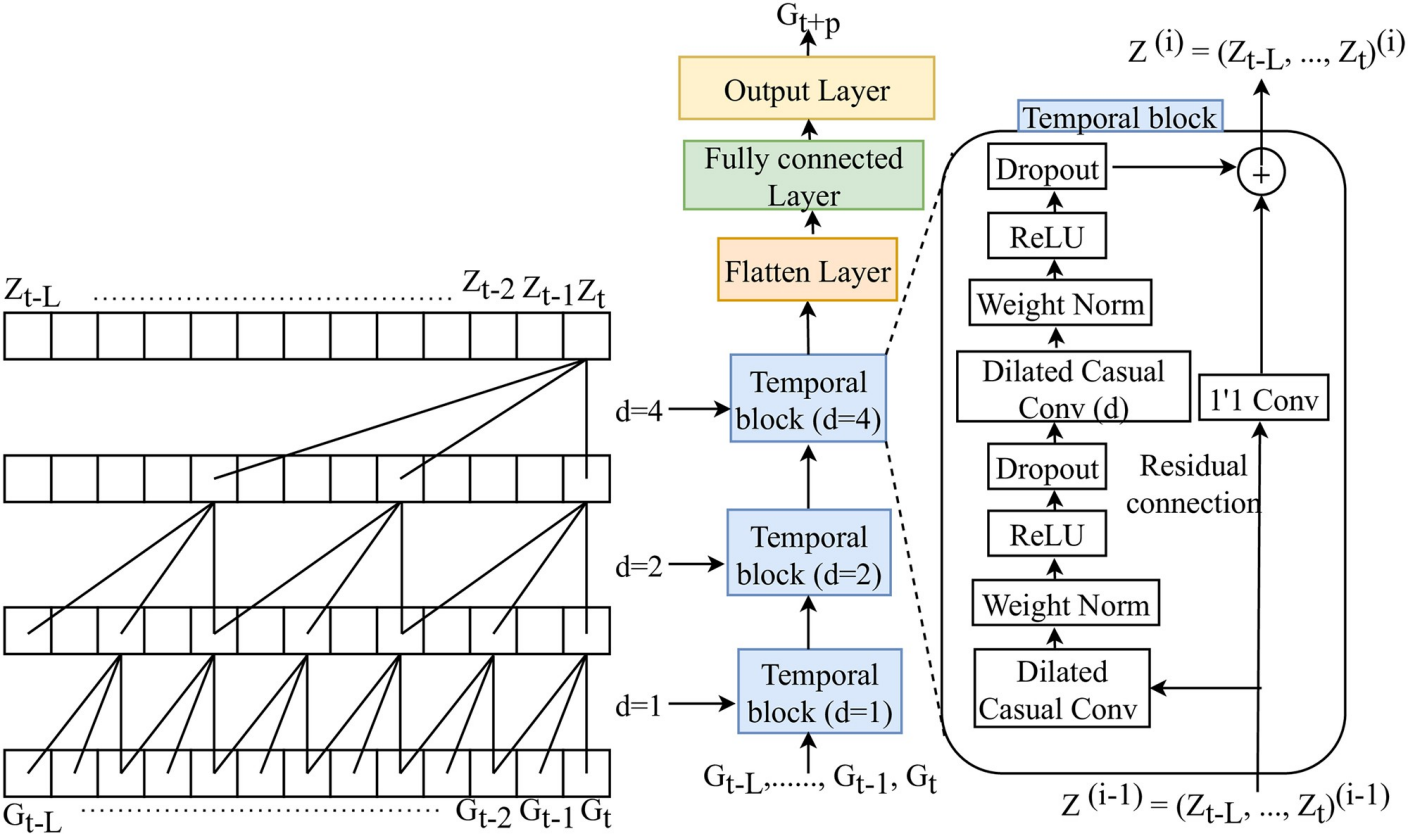
The schematic plot of TCN, where left figure shows the structure of dilated causal convolution with dilation d = 1,2,4. (middle) Framework of TCN for BGL prediction that includes temporal residual blocks with different dilations and a fully connected layer connected to the last layer. (right) The internal structure of the temporal residual block.

A temporal convolutional network (TCN) is a convolutional neural network that handles time series data [34]. It can examine long-range patterns using a hierarchy of temporal convolutional filters [35]. Dilated convolutions harnessed within TCNs expand the receptive field exponentially across each layer, capturing long-range data dependencies within the data [36] and utilizing residual connections, facilitating the more accessible training of deep models [8] as shown in Fig 3.

Given their ability to address extensive dependencies within time series data, TCNs are particularly instrumental for predicting BGL. BGLs, responsive to factors manifesting hours or even days prior, necessitate models capable of learning such extended dependencies—a capability TCNs possess. Few studies have considered TCN for BGL prediction [8, 37]; nevertheless, these networks have successfully applied for other time series forecasting tasks [19]. Thus, TCN is considered pivotal for the comparison in this study.

### Long-short-term memory neural network

**Fig 4:**
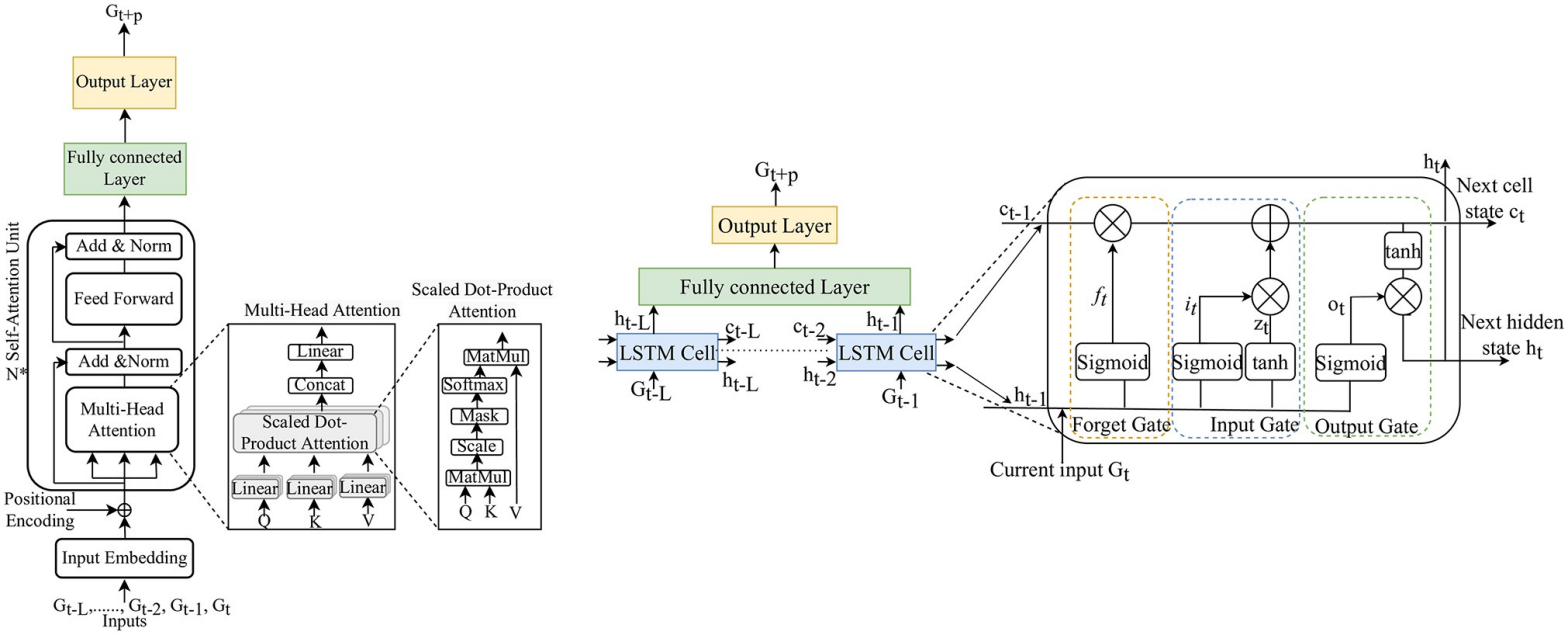
The schematic plot of (Left) SAN, (left) the transformer encoder architecture with N-self-attention units connected to a fully connected layer to predict BGLs. (middle) Multi-head attention within the encoder consists of several attention layers running in parallel. (right) The internal structure of scaled dot product attention comprises dot product computation, scaling, and application of softmax. (Right) LSTM network. (left) An LSTM network with an LSTM layer, and fully connected layer to predict BGL. (right) The internal architecture of the single LSTM cell with input, cell, and hidden state with activation function.

Long short-term memory (LSTM) networks are a specialized variant of recurrent neural networks (RNNs) designed to capture extensive temporal dependencies within sequential data [3]. The LSTM comprises memory cells, input, forget, and output gates that dynamically regulate information flow, preserving the critical patterns and insights across prolonged sequences, making it more effective for time series prediction [9]. Each LSTM cell has two states that are passed from the current to the next step, cell state *c*_*t*_ and hidden state *h*_*t*_, which is used to compute the output, as illustrated in the right of Fig 4.

LSTM models exhibit multiple layers of recurrent units that share identical parameters and incorporate loops propagating the data back to the same computation units, considering the current input and the knowledge acquired from prior inputs, making it suitable for time-series predictions [3]. This intrinsic mechanism provides LSTMs exceptional capabilities in capturing complex temporal correlations and excelling in diverse time series forecasting problems [38]. Such capacity is pivotal for achieving precise prediction accuracy, especially given the substantial variability and trends often seen in BG trajectories over extended periods. Therefore, LSTM is the most widely used algorithm for BGL prediction or any other time series prediction task [3, 7–9, 12, 19, 24, 32]. Study [8] presents an experimental comparison between classical regression models and deep learning models. The evaluation considered different input features, regression model orders, and methods for multi-step prediction of blood glucose (BG) levels (recursive vs. direct), revealing no significant advantage of machine learning models over classical ones. Although the study [8, 12, 15] provided valuable insights and improved performance, they lack analysis across diverse patient groups. Similarly, the study [3] utilized larger datasets with heterogeneous sets of patients and obtained improved performance with Tikhonov regularization, the study lacks thorough validation with diverse patient groups. Without such validation the generalizability cannot be confirmed.

### Self-attention network

A self-attention neural network, often associated with the transformer architecture, leverages attention mechanisms to integrate input features or sequences, focusing on the most critical information for the specific task [39], as shown in Fig 4 (left). In the context of time series prediction, a self-attention neural network is designed to learn temporal dependencies and patterns within the data, giving attention to specific time points or intervals [10]. In the self-attention mechanism, each element or token in the input sequence is correlated to a set of query, key, and value vectors learned through the training process [39].

Originally discovered for natural language processing (NLP) and computer vision applications, numerous attention mechanisms have been proposed, with the transformer being successfully implemented in NLP [40]. In the context of BGL prediction, [5, 10, 11, 13] exploited attention mechanism-based networks. In the work [10], SAN was implemented and assessed for its performance, which we considered for our investigation. The study [10, 11] explored attention-based deep networks and enhanced personalized prediction. Similarly, an attention-based recurrent network for personalized blood glucose level predictions, incorporating model confidence through an evidential layer, showed improved performance in [13]. Although these models utilized diverse datasets with larger sample sizes, their analysis is limited in terms of robustness.

Overall, the comparison in the table and the description demonstrates that most studies rely on limited sample populations or data collected over brief periods. Even when multiple datasets are used in the analyses, the models’ generalizability—a crucial factor for practical application in diverse real-world clinical settings—has not been evaluated. To overcome these limitations, this study evaluates the performance of different models across various patient groups, examining their applicability and generalizability in diverse contexts. This approach ensures the models’ reliability in real-world diabetes care, which encompasses a wide range of patients with differing conditions and blood glucose dynamics.

## Methods

The BGL prediction problem is to estimate the future BGL of diabetes patients at different short- and long-term PHs, given a sequence of BGLs measured by CGM at each time interval. The BGL data are obtained from a real-time CGM sensor, measured every 5-minute interval, and are the primary driver of the BGL prediction algorithm. Given these input drivers, the sequence of BGLs (*G*_*t*_, *G*_*t*−1_,…*G*_*t*−*L*_) at time *t* with window length *L*, and a PH *p*, the aim is to predict BGL *G*_*t*+*p*_ at time *t* + *p*. For this prediction, data-driven models are trained using the BG data collected from a large number of patients and collected over a long time. The final trained model is utilized in BGL prediction. The BGL predicted at time *t* + *p* is given by:

$$\begin{array}{cc} {\hat{G}}_{t+p}=M(G_{t},G_{t-1}, & \ldots G_{t-L}) \end{array}$$ (1)

where *M* is the model that takes a sequence of BG values of window size *L*, i.e. time steps or historical data, as input to the model.

The proposed study is to assess the obtained BGL predictive models based on different datasets to gauge their capabilities for their application in real-world scenarios. Different evaluation metrics that compare the predicted ${\hat{G}}_{t+p}$ and ground truth BG values *G*_*t*+*p*_ are utilized to assess the models.

Over the past two decades, numerous algorithms for predicting future glucose levels have been developed using CGM data alone [3, 5, 10, 11, 27, 28, 33] and in combination with other data like carbohydrate intake, insulin, and physical activity [8, 12, 29]. Although additional data might improve predictions, they require extra devices and actions, making CGM-only algorithms crucial due to their practical usability and current underused complex systems. Also, some studies have shown high accuracy only with the inclusion of CGM data [27, 28]. Thus, for the comparison, only the data on BG is integrated into this study. The analysis is on the four distinct datasets, OhioT1DM, DCLP3, DCLP5, and RT, of varying sample counts and diverse characteristics incorporating age range, sample size, and duration of sample collection, as outlined in Table 2. Also, the diversity contributes to the series of BG dynamics envisioned within each dataset. For instance, two datasets include sample data from patients undergoing insulin therapy using a closed-loop system, offering precise control over BGLs. A closed-loop system automates insulin delivery by continuously monitoring BGLs and regulating insulin infusion rates in real-time. The presence or absence of this control system influencing the datasets presents diverse glucose dynamics, a crucial factor in training a model to excel in generalization. Consequently, this study aims to realize a model that can be employed universally across different contexts, leveraging the diverse dynamics in the sample data from various datasets. We anticipate that creating a robust and versatile model is valuable in the practical context of BGL prediction, where different factors like meals and physical activities influence BGLs within diverse patients groups.

**Table 2:** Summary of datasets with respective sample counts.

	Ohio	DCLP3	DCLP5	RT
**No. of Patients**	12	112	101	451
**Age range (years)**	20-80	14-71	8-13	above 8
**Duration**	8 weeks	6 months	28 weeks	26 weeks
**Under therapy (closed loop system)**	No	Yes	Yes	No
**Original**	166532	9032235	3780007	710838
**Resampled**	188983	9057472	3790618	826813
**Training**	170387	5462133	2345757	487468
**Testing**	8441	1827016	793177	158541
**Validation**	10155	1768323	651684	180804

### Dataset description

#### OhioT1DM dataset

The OhioT1DM [20] dataset, encompasses eight weeks of data for each of the twelve patients having type-I diabetes, where data of six patients were released in 2018 in the first BGL prediction challenge, and another six were released in the second challenge held in 2020. The dataset encompasses seven males and five females within the age range of 20 to 80 years. Data collection involved data collected every five minutes using Medtronic 530G insulin pumps and Medtronic Enlite CGM sensors, supplemented by other daily events reported by the patients via a smartphone app or the fitness band. This study explores only the data on BGLs. Table 2 summarizes the dataset’s characteristics regarding age range, duration of sample collection, sample size, and number of training and test samples.

#### DCLP3 dataset

The DCLP3 [23] is a publicly available dataset from a 6-month randomized, multicenter trial. The dataset includes 112 T1D patients who used Tandem t: slim X2 with Control-IQ Technology [41] and Dexcom G6 CGM for diabetes management [42]. The data contributors were under closed-loop control system-based insulin therapy and were 14 to 71 years old, including both adolescents (14-19 years old) and adults (above 20 years). BGLs were recorded approximately every five minutes during six months for each subject. The characteristics of the dataset are depicted in Table 2.

#### DCLP5 dataset

The DCLP5 [22] consists of BG monitoring data from 101 children, aged 6 to 13 years and having type I diabetes, collected from a multicenter randomized trial over 16-week period. The trial was to assess the effectiveness of the closed-loop system; thus, all the data was collected from the contributors who underwent insulin therapy using a closed-loop system. The dataset contains BGLs monitored via a Dexcom continuous glucose monitoring device. The characteristics of the dataset are described in Table 2.

#### RT dataset

The publicly available RT [21] is a comprehensive dataset that encompasses the BGLs of 451 diverse type-1 diabetes patients, rigorously randomized to ensure a fair representation. The patient cohort comprises a balanced mix of genders (45% male and 55% female) across three age groups: adults (above 20 years old), adolescents (14-19 years old), and children (8-13 years old). The dataset comprises glucose level measurements captured every five minutes using three different CGM devices: DexCom [42], Abbott Diabetes [43], and Medtronic [44].

**Fig 5:**
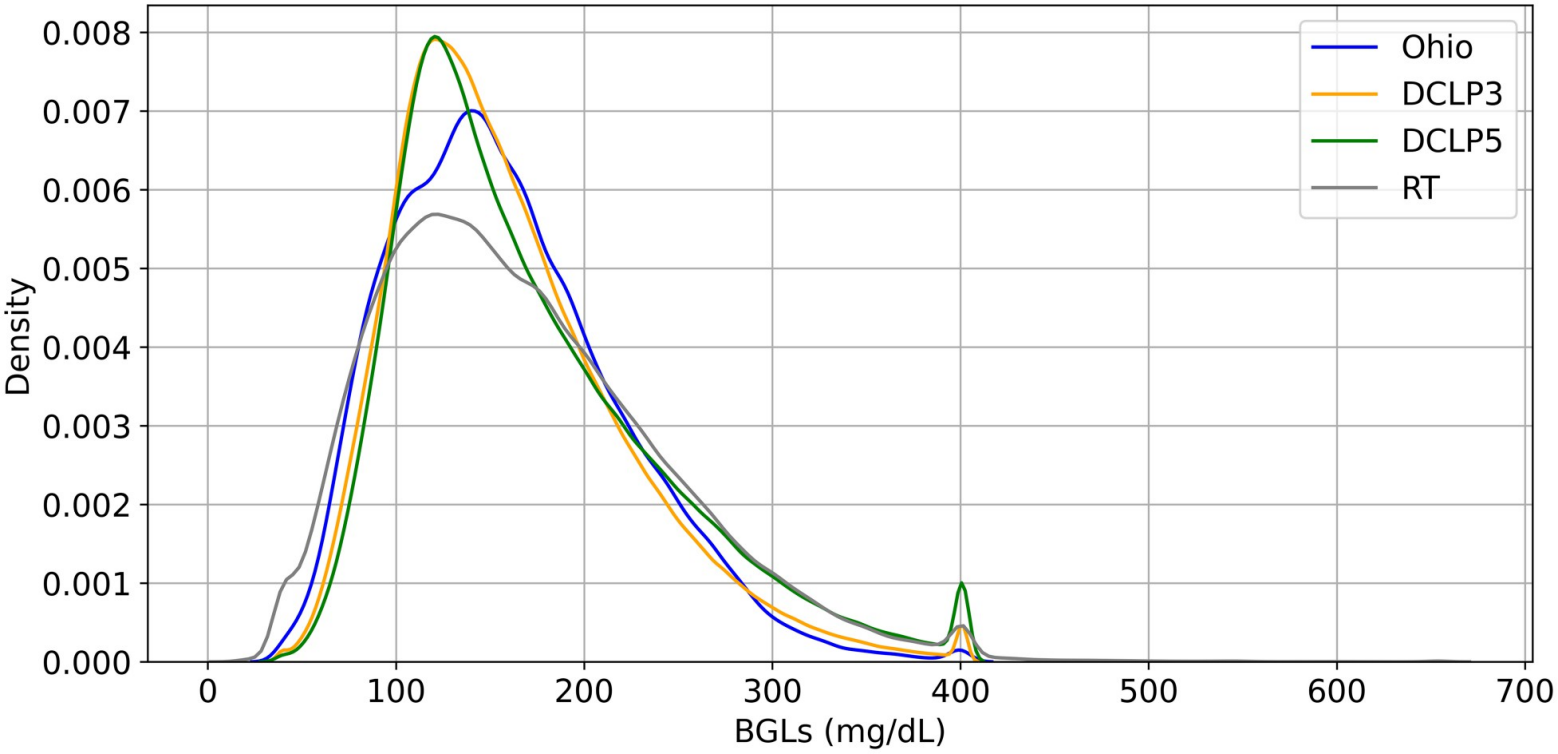
A visual representation of data distribution across each dataset, showcasing where data spreads out, or are more concentrated, and how they are distributed across the range of possible values.

Since the central focus of this study is to evaluate the models’ behavior and their generalization capabilities across datasets with varied BG dynamics, the kernel density estimation plot is presented in Fig 5 to observe the underlying distribution and variability across all the datasets. The plot shows two prominent peaks, suggesting all the datasets follow a bimodal distribution. The first peak occurs around BGLs 100- 200 mg/dl for all datasets, with a significant portion of data points in each dataset are concentrated around this BG ranges. DCLP5 and DCLP3 have the most consistent and similar distribution, while the distribution of Ohio is the broadest of the four, with the least consistent distribution and significantly more variations in data. Dataset RT falls between, having slightly more variations in data, meaning it has lower data consistency than DCLP3 and DCLP5. The second peak is around BGLs 400 mg/dl for all datasets, another significant concentration of data points in this range. The height of this peak is lower than the first, suggesting fewer data points are concentrated here compared to the first peak. The densities are more closely aligned among all datasets, with RT having higher density among all datasets.

### Data preprocessing

The preprocessing stage includes handling missing data, standardizing it to a common scale for generalization and simplified analysis. Due to data collection from multiple devices or device errors, there were instances of missing data in each dataset. Therefore, a two-stage data cleaning approach was implemented: resampling and data imputation. The resampling stage aligns all data to the same time interval, while the imputation stage fills in the missing gaps.

#### Resampling

Resampling is a preprocessing step that allows the adjustment of the data’s frequency to match regular time series of specific time intervals without missing data. A 5-minute time grid was established for this study to align with the 5-minute sampling interval of the BG sensor data across training sets of all datasets covering the entire BG signal duration, and to insert data points within missing gaps. All accumulated signals were aligned with this time grid, and the missing values were filled using a “linear interpolation” approach. In this approach, missing values in data are interpolated by fitting a straight line between existing data points. This study discarded data consecutively missing for over an hour to avoid artifacts and incorrect prediction trajectories.

#### Normalization

Data normalization is a data preprocessing technique that transforms numerical data into a standard scale, which makes it easier for different data sets to be compared and analyzed. Standardized normalization is employed in this study, transforming numerical data into a standardized scale with a mean of 0 and a standard deviation of 1. Standardization involves subtracting the mean from each data point and dividing it by the standard deviation, as in (2).

$$\begin{array}{c} Z_{t}=\frac{G_{t}-\mu }{\sigma } \end{array}$$ (2)

where, *G*_*t*_ and *Z*_*t*_ denotes the original and normalized BG value at time *t*. *μ* refers to the mean value of the BG data with *n* number of observations and *σ* represents the standard deviation.

Since the subjects within each dataset do not exhibit different ranges of values and the focus of this study is not on personalized models, normalization is applied to each dataset as a whole. The normalization parameters *μ* and *σ* computed from the training dataset are used to normalize training, validation, and test datasets, resulting in a new set of data points where the mean of the data points is 0, and the standard deviation is 1.

### Hyperparameter tuning and model training

Hyperparameter tuning for each model was conducted using the grid search method, with the respective hyperparameters listed in Table 3. The optimal hyperparameter configuration, which minimized the model’s loss function, was identified to yield the most favorable model performance. The hyperparameters for SAN, such as the number of attention layers, number of attention heads, and dimension of hidden neurons, however, were selected based on the literature study [5]. Since this network is already been validated and successfully implemented, leveraging such architecture, particularly those with deep structures, can mitigate the risk of overfitting. Thus, the network is configured with 3 attention layers, each containing 4 attention heads, and hidden dimensions of 128 set to the multi-head attention module and 512 to the feed-forward layer. Tuning of other hyperparameters, such as learning rate, batch size, and dropout, was carried out using grid search methodology as is done for other network architectures. Owing to the varied data size of each dataset, batch size was distinct for each of them; 1024 was the optimal size for DCLP3 and DCLP5, while for RT, 512 was the ideal size, and for OhioT1DM, 16 was the best fit. Further, CNN and TCN obtained optimum performance with a batch size of 16 for every dataset.

**Table 3:** Hyperparameter tuning and optimal configuration.

Model	Hyperparameters	Range	Optimal Value
**FNN**	Number of layers	1-4	2
	Number of neurons	16,32,64, 28	32,64
	Dropout	0.1-1	0
	Learning rate	0.01-0.00001	0.01
	Batch size	16-1024	16, 512, 1024
**CNN**	Number of layers/ FC layers	1-4	2/2
	Number of neurons/ FC neurons	16,32,64,128	32, 64/ 64,32
	Dropout	0.1-1	0
	Learning rate	0.01-0.00001	0.001
	Batch size	16-1024	16
**TCN**	Number of layers/ FC layers	4-10	4/2
	Number of neurons/ FC neurons	16,32,64,128	16,32,64/ 64
	Dropout	0.1-0.5	0
	Learning rate	0.01-0.00001	0.001
	Batch size	16-1024	16
**LSTM**	Number of hidden units	30,50,100,300	30,30
	Dropout	0.1-0.5	0
	Learning rate	0.01-0.00001	0.001
	Batch size	16-1024	16, 512, 1024
**SAN**	Learning rate	0.01-0.00001	0.001
	Dropout	0.1-0.5	0
	Batch size	16-1024	16, 512, 1024

Each optimized model was trained using each dataset, incorporating a hold-out set cross-validation approach to mitigate overfitting risks. Each dataset was initially segmented into training and test sets, comprising 80% and 20% of the data. The training set was further subdivided into an 80% training subset and a 20% hold-out validation subset. The hold-out validation subset facilitated hyperparameter tuning, while the primary training subset was utilized to train the models. The model’s performance was evaluated on the test set, which represented unseen data for the model. Notably, the data partitioning was conducted chronologically to ensure that the hold-out validation and test sets exclusively comprised data instances not present in the training subset. Also, patient allocation ensured complete separation between the training and test datasets, meaning the data from any specific patient can appear in either the training or test set. Since the OhioT1DM dataset originally included testing and training data for each of the 12 patients, those data are used as is for training and testing.

For the realization in the practical scenario, a sliding window of 2 hours of historical data is utilized as input to the model to predict the BGL, 30 minutes and 60 minutes in advance. Each model underwent training for 100 epochs across all datasets with five repetitions for each training run, where the root mean squared error (RMSE) is used as a cost function. The adaptive moment estimation (Adam) optimizer [45], appropriate for non-stationary data like BG, is employed as an optimizer to minimize the RMSE, and for each epoch, average RMSE loss over all batches is utilized to update the model parameters. The best model with the lowest RMSE value is stored for the performance evaluation with the unseen test data. The optimization parameters including learning rate decay of 0.1, decay patience of 10, and patience for early stopping of 30, were utilized for each model training. All the assessments were performed with a completely new and unseen test set from each dataset. The effectiveness of each model was evaluated by comparing the predicted BG value ${\hat{G}}_{t+p}$, forecasted *p* minutes in advance, with the corresponding ground truth value *G*_*t*+*p*_. To estimate the generalization capacity of each model, the model trained with one dataset underwent evaluation using the test set of other datasets. The model development, hyperparameter tuning, training, and testing were conducted within Jupyter Lab using Python programming language and Pytorch.

### Performance evaluation criteria

#### Analytical assessment

To assess the performance of each model, four different regression-wise metrics that quantify the similarity between predicted and reference BG values are implemented. These are the most widely used metrics to assess the accuracy of BGL prediction. The computation is carried out for each prediction horizon separately.

*RMSE*. Root mean square error is the prediction error, defined as the standard deviation of the difference between the predicted and reference ground truth value as is given in (3). The smaller the RMSE value, the more reliable and accurate is the predictive model. In this study, RMSE is utilized as a cost function for the optimization and as a measure for evaluating the models during training.

$$\begin{array}{c} RMSE=\sqrt{\frac{1}{N}\sum_{i=1}^{N}{\left(G_{i}-{\hat{G}}_{i}\right)}^{2}} \end{array}$$ (3)

Here, *i* is an index that runs from 1 to *N*, representing each individual reference point in the test set. *G*_*i*_ and ${\hat{G}}_{i}$ are the *i*th reference and predicted value, and *N* is the test dataset size.

*COD*. COD or R2 is the Coefficient of Determination, defined as the square of the correlation (R) between predicted and reference values, that compares the variance of the model’s predictions to the variance of reference values. Thus, it ranges from 0 (absence of correlation) to 1 (complete correlation), with values towards zero indicating performance degradation, while values close to 1 suggest better performance. The COD is calculated as follows:

$$\begin{array}{c} R^{2}=1-\frac{\sum_{i=1}^{N}{\left(G_{i}-{\hat{G}}_{i}\right)}^{2}}{\sum_{i=1}^{N}{\left(G_{i}-\bar{G}\right)}^{2}} \end{array}$$ (4)

where *G*_*i*_,${\hat{G}}_{i}$, $\bar{G}$ and *N* are the reference value, predicted value, mean value of reference data, and the test dataset size.

*MAD*. MAD is the Mean Absolute Difference between predicted and observed values. A lower MAD value indicates superior performance, while the performance is considered poor if the value is high. MAD value is computed as:

$$\begin{array}{c} MAD=\frac{1}{N}\sum_{i=1}^{N}\left|G_{i}-{\hat{G}}_{i}\right| \end{array}$$ (5)

where *G*_*i*_,${\hat{G}}_{i}$, and *N* are the reference value, predicted value, and the test dataset size.

*FIT*. FIT is computed as the ratio of RMSE, and the root mean square difference between the reference value and its mean value as in the equation:

$$\begin{array}{c} FIT=(1-\frac{\sqrt{\frac{1}{N}\sum_{i=1}^{N}{\left(G_{i}-{\hat{G}}_{i}\right)}^{2}}}{\sqrt{\frac{1}{N}\sum_{i=1}^{N}{\left(G_{i}-\bar{G}\right)}^{2}}})\times 100 \end{array}$$ (6)

where *G*_*i*_,${\hat{G}}_{i}$, $\bar{G}$, and *N* are the reference value, predicted value, mean value of reference data, and test dataset size. It represents the measure of improvement or reduction in error compared to a baseline model that predicts the mean. Thus, the FIT value close to 100% indicates better performance, while a lower value suggests the model’s performance is closer to that of baseline mean prediction.

*MADP*. Next, to quantify the error in a standard scale, mean absolute difference percentage (MADP), an average percentage deviation between predicted and actual values used. Since MADP normalizes errors by the actual values, it expresses errors in a single scale as a percentage of the actual values and provides a more interpretable measure of relative deviation as given in (7). Unlike RMSE, which penalizes large errors more heavily, MADP treats all errors equally. The lower MADP value suggests better accuracy and closer alignment between predicted and actual values, while the higher MADP indicates a higher percentage difference between the two.

$$\begin{array}{c} MADP=\frac{1}{N}\sum_{i=1}^{N}(\frac{|G_{i}-{\hat{G}}_{i}|}{G_{i}})\times 100 \end{array}$$ (7)

where *G*_*i*_,${\hat{G}}_{i}$, and *N* are the reference value, predicted value, and the test dataset size.

#### Clinical assessment

Although regression analysis can provide overall insights into predictive performance, it often falls short in identifying crucial outliers and providing clinical interpretation leading to inaccurate treatment decisions. Thus, Clarke Error Grid (CEG) analysis [46] is utilized to give a more holistic assessment of the model’s performance. The CEG is visualized through a scatterplot divided into five regions, which maps predicted and reference BGLs.

**Region A** predicted values lie within 20% of the reference value.
**Region B** The predicted value lies beyond 20% of the reference value but is considered clinically non-threatening.
**Region C** predicted values might cause inappropriate treatment without any dangerous consequences to the patient.
**Region D** predicted values indicating potentially dangerous failure to detect any events.
**Regions E** predicted values indicating the opposite treatment initiatives, treatment of hyperglycemia instead of hyperglycemia and vice versa.

#### Assessment of generalization ability

We further analyzed how well the models can extend their performance to completely unfamiliar datasets to obtain valuable insights into the model’s generalization capabilities and robustness across diverse datasets. For this, the model initially trained on one dataset was tested with different datasets and RMSE was used for the evaluation.

Next, we conducted a statistical test to assess the generalizability of the models across datasets, determining whether the models are statistically consistent across datasets. We performed a Kolmogorov-Smirnov (KS) test on residuals of a model trained on one dataset and tested with other datasets to determine the distribution differences in residuals across datasets. While RMSE is directly related to prediction accuracy and is crucial for comparing different models or assessing the model’s performance, residuals are the difference between actual values and predicted, it can identify trends and biases along with the limitations in model’s prediction [47, 48]. It is useful for assessing whether the model’s assumptions are valid across different datasets. Thus, instead of using RMSE distributions, residuals are considered for this statistical analysis. If the distribution of residuals is consistent across datasets, it indicates that the model performs and generalizes to the unseen data. Thus, the null hypothesis result for two residual distributions generated by testing the model with two different datasets, unseen during training, implies that the distribution of residuals is not significantly different. Whereas the alternative hypothesis suggests that the distribution of residuals is significantly different. The significance threshold of 0.05 was considered for this test.

## Experimental results and discussion

### Analytical assessment

Table 4 outlines the performance of each model, trained with four distinct datasets, for PHs of 30 and 60 minutes. The results presented are the mean values for the respective metrics. All models demonstrated varying performance, exhibiting higher accuracy (lower RMSE) for datasets Ohio and DCLP3 in comparison to DCLP5 and RT for both short- and long-term predictions. Notably, the models for DCLP5 and RT exhibited similar performance. Among all the models, LSTM achieved good performance across all datasets and PHs, with the lowest RMSE and MAD values and the highest FIT and COD values, followed by SAN exhibiting comparable performance. FNN emerged as the least performing model among the entire models across all datasets for both short- and long-term prediction, though the models TCN, CNN, and FNN performed relatively similarly with closer prediction accuracy. When considering the FIT values against the baseline model, models with dataset Ohio consistently outperformed others, with both DCLP3 and RT presenting FIT values relatively close yet lower than Ohio, followed by DCLP5.

**Table 4:** Predictive performance (RMSE, MAD, COD, and FIT values) of each model across each dataset for PH 30min and 60min.

		FNN		LSTM		SAN		CNN		TCN	
Dataset	Metrics	30min	60min	30min	60min	30min	60min	30min	60min	30min	60min
**Ohio**	**RMSE (mg/dL)**	22.12 ± 0.24	35.19 ± 0.36	18.26 ± 0.25	31.12 ± 0.35	18.67 ± 0.26	32.08 ± 0.34	20.49 ± 0.28	32.4 ± 0.35	20.54 ± 0.26	32.57 ± 0.35
	**MAD (mg/dL)**	15.69 ± 0.16	25.9 ± 0.25	12.15 ± 0.14	21.83 ± 0.23	12.4 ± 0.14	22.23 ± 0.14	14.43 ± 0.15	22.9 ± 0.23	14.23 ± 0.15	23.29 ± 0.25
	**COD**	0.86	0.65	0.9	0.72	0.92	0.71	0.9	0.7	0.87	0.7
	**FIT (%)**	66	41	69	47	68	46	65	35	65	45
**DCLP3**	**RMSE (mg/dL)**	25.381 ± 0.05	38.81 ± 0.06	20.33 ± 0.05	34.91 ± 0.05	20.63 ± 0.05	35.23 ± 0.06	23.51 ± 0.05	38.82 ± 0.06	25.45 ± 0.05	38.03 ± 0.05
	**MAD (mg/dL)**	17.9 ± 0.03	28.52 ± 0.04	13.84 ± 0.02	25.59 ± 0.03	14.07 ± 0.02	25.64 ± 0.03	16.64 ± 0.02	28.68 ± 0.04	18.55 ± 0.03	28.42 ± 0.04
	**COD**	0.81	0.57	0.88	0.65	0.88	0.64	0.84	0.56	0.81	0.58
	**FIT (%)**	57	34	65	41	65	40	60	34	57	35
**DCLP5**	**RMSE (mg/dL)**	32.17 ± 0.09	47.22 ± 0.10	26.38 ± 0.09	43.66 ± 0.10	26.82 ± 0.08	43.98 ± 0.10	29.28 ± 0.13	44.81 ± 0.26	30.26 ± 0.08	44.97 ± 0.10
	**MAD (mg/dL)**	22.9 ± 0.05	35.17 ± 0.07	18.5 ± 0.04	32.17 ± 0.06	18.97 ± 0.04	31.71 ± 0.07	20.81 ± 0.07	32.73 ± 0.18	21.79 ± 0.05	33.49 ± 0.07
	**COD**	0.77	0.51	0.85	0.58	0.84	0.58	0.81	0.56	0.8	0.56
	**FIT (%)**	52	30	61	35	60	35	57	34	55	33
**RT**	**RMSE (mg/dL)**	29.85 ± 0.25	45.21 ± 0.27	26.31 ± 0.26	41.94 ± 0.27	26.7 ± 0.27	42.38 ± 0.27	28.32 ± 0.25	43.25 ± 0.27	29.08 ± 0.24	44 ± 0.27
	**MAD (mg/dL)**	20.25 ± 0.10	32.72 ± 0.16	16.68 ± 0.10	29.31 ± 0.14	16.99 ± 0.10	30.16 ± 0.15	18.78 ± 0.10	30.45 ± 0.15	19.57 ± 0.11	31.17 ± 0.14
	**COD**	0.84	0.64	0.88	0.69	0.87	0.68	0.86	0.67	0.85	0.66
	**FIT (%)**	60	40	65	44	64	44	62	42	61	41

**Fig 6:**
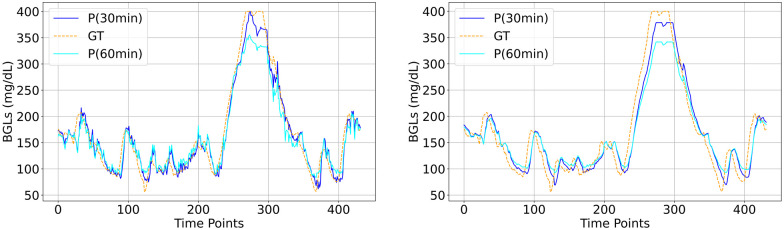
BG trajectory plot of predicted (P) vs ground truth (GT) of 1.5 days for both PHs obtained with (Left) best (LSTM) and (Right) worst (FNN) models trained with Ohio.

**Fig 7:**
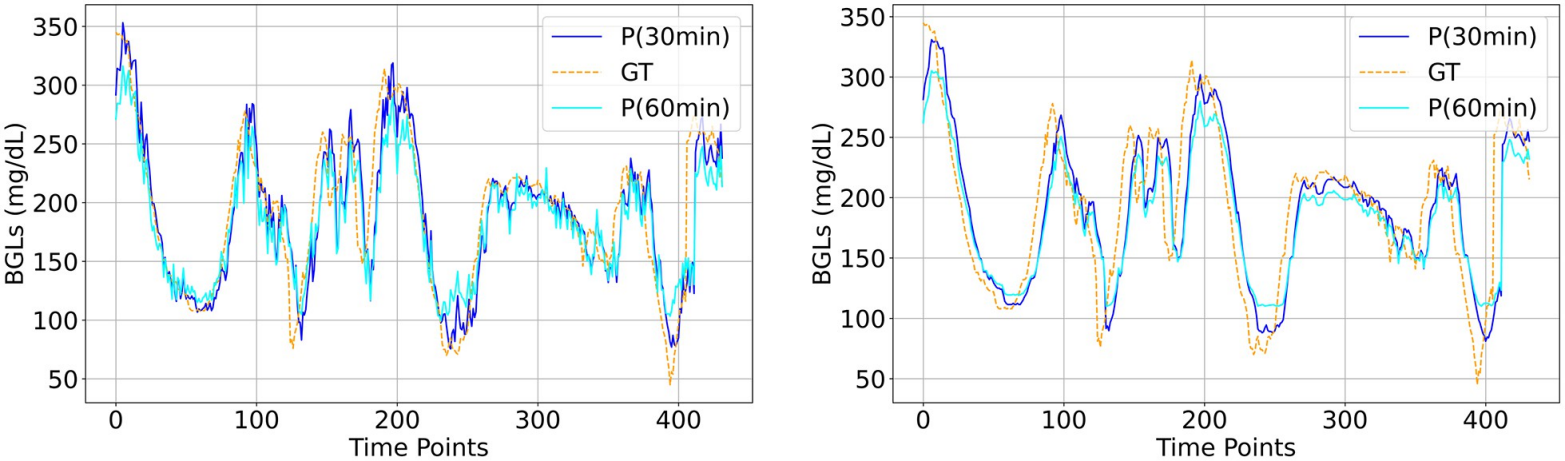
BG trajectory plot of predicted (P) vs ground truth (GT) of 1.5 days for both PHs obtained with (Left) best (LSTM) and (Right) worst (FNN) models trained with DCLP3.

**Fig 8:**
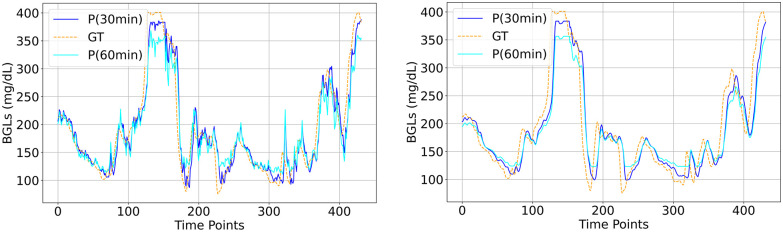
BG trajectory plot of predicted (P) vs ground truth (GT) of 1.5 days for both PHs obtained with (Left) best (LSTM) and (Right) worst (FNN) models trained with DCLP5.

**Fig 9:**
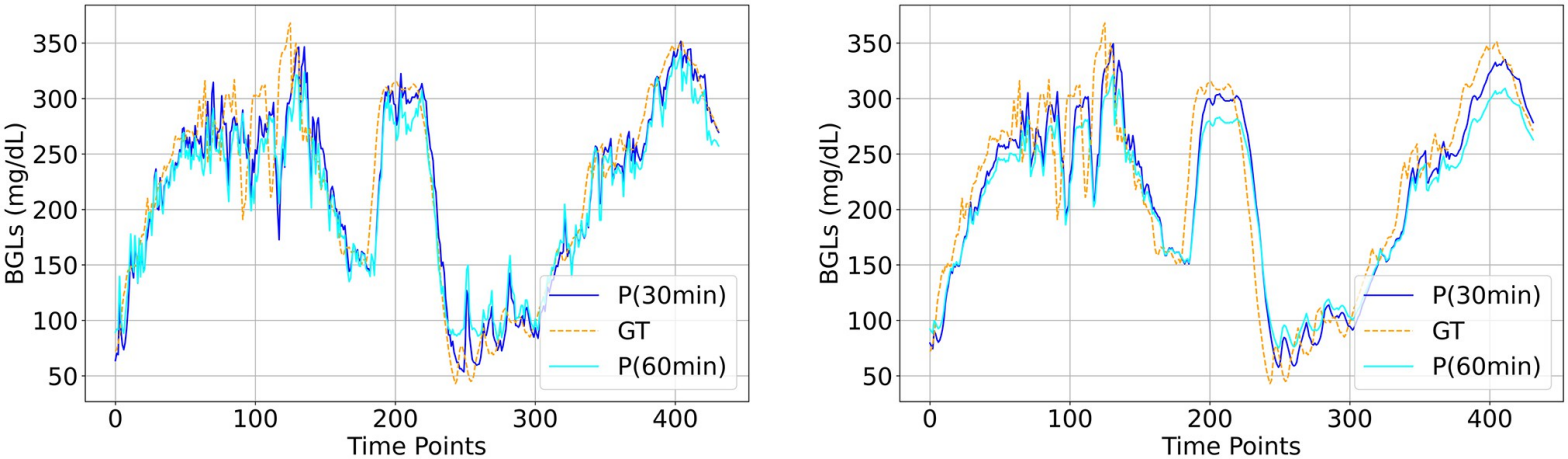
BG trajectory plot of predicted (P) vs ground truth (GT) of 1.5 days for both PHs obtained with (Left) best (LSTM) and (Right) worst (FNN) models trained with RT.

**Fig 10:**
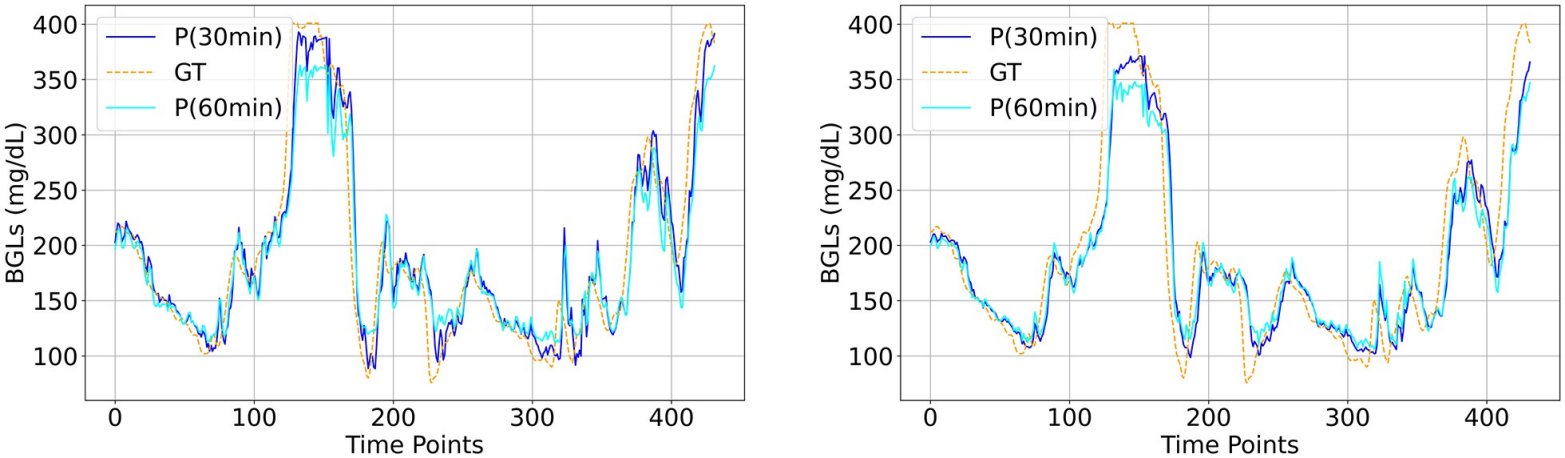
BG trajectory plot of predicted (P) vs ground truth (GT) of 1.5 days for both PHs obtained with (Left) SAN and (Right) CNN trained with DCLP5.

The visual plots of predicted vs actual BGLs in Figs 6–10 demonstrate that the LSTM and SAN have minimal errors at peaks and troughs compared to FNN within hyperglycemia and hypoglycemia regions. 60-minute predicted trajectory follows the patterns of the 30-minute trajectory except around those peaks and troughs regions with higher discrepancies. Besides, trajectories of predictions obtained from LSTM and SAN are more fluctuating than the results obtained from other models, with more fluctuations occurring around where the ground truth signal is more oscillating. Even though the error is higher with FNN within the entire signal, its prediction trajectory follows the pattern of the ground truth curve suggesting that the model is able to capture the patterns and trends of the ground truth data. Further, inspecting performance for the datasets, for Ohio and DCLP3, models LSTM and SAN obtained minimal errors along hyper-hypo regions, while the error is more pronounced in DCLP5 and RT. As observed as a worst-performing model among the five in Table 4, FNN’s performance is worst around every region including the highest and lowest peaks compared to other regions.

**Fig 11:**
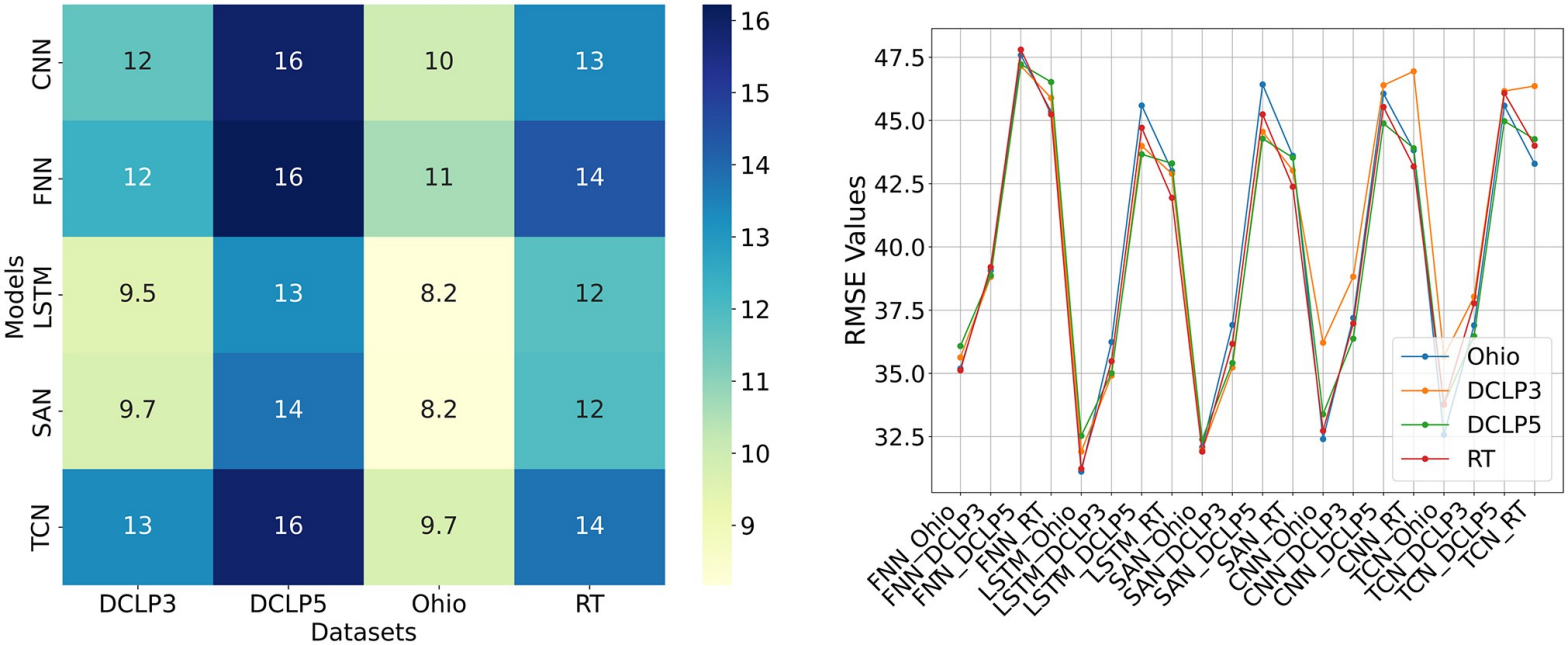
(Left) Visual plot of a heatmap of average percentage deviation between predicted and actual values, defined as MADP, of each model for all four datasets. (Right) Visual plot of RMSE values obtained with models trained on one dataset and tested with the other. X-axis represents the models with test dataset, while the dataset in the legends indicate the corresponding train datasets used to train those models.

Next, we analyzed the results based on MADP, as shown in Fig 11 in left. Each cell in the heatmap represents the MADP value for the corresponding model and dataset. LSTM has the lowest MADP value across all datasets suggesting a better predictive accuracy, while FNN has the highest MADP value for the majority of datasets, indicating itself as a low-performing model. Dataset Ohio seems to be more compatible with the models yielding consistently lower MADP values over all the models. In terms of consistency across datasets, all models show variability in the performance, with models SAN and LSTM demonstrating relatively stable performance across datasets with lower MADP values than others. This result suggests that the models LSTM and SAN might be applicable where consistency across different datasets is crucial.

### Clinical assessment

Next, clinical validation is carried out using CEG analysis, the result of which is presented in Table 5. Each column represents the result of CEG for different PHs, giving a percentage of the predicted BG values that fall within the specific zone. For better understanding, five zones of CEG are merged into two zones, zones A and B are combined to represent the clinically safe prediction zone, while zones C, D, and E, which are considered unsafe, are merged to represent unsafe predictions. All the models achieved satisfactory results, with more than 91% of the predictions falling in the safe region for both PHs. Yet again, LSTM, and SAN achieved the highest clinical accuracy across all datasets for both PHs, with CNN exhibiting similar performance to SAN for long-term prediction. They exhibited higher performance with Ohio and conversely, lower performance with RT, with a decreasing trend as the PH extended. On the other hand, FNN demonstrated a close resemblance to TCN for both PHs, showcasing more consistent and higher accuracy on short-term prediction compared to long-term predictions. Besides, a relatively higher percentage of predictions obtained from models trained with RT lies within unsafe regions compared to other datasets. 96% of predictions lie within (A+B) regions for FNN with Ohio (for 60-minute PH) while only 92% of predictions lie within that region for dataset RT. Thus, overall, the top-performing model is LSTM, consistently excelling within every dataset, while FNN emerges as the least-performing model, especially when trained with dataset RT.

**Table 5:** Results of CEG for five models trained across four datasets for PHs (30 and 60 minutes).

		30min		60min	
Model	Dataset	A+B (%)	C+D+E (%)	A+B (%)	C+D+E (%)
**FNN**	**Ohio**	98.51	1.49	96.21	3.78
	**DCLP3**	98.16	1.83	96.67	3.32
	**DCLP5**	97.36	2.63	95.04	4.95
	**RT**	97.17	2.83	91.67	8.32
**LSTM**	**Ohio**	99.42	0.58	96.74	3.25
	**DCLP3**	98.77	1.23	97.46	2.53
	**DCLP5**	98	1.99	96.05	3.96
	**RT**	98.23	1.76	93.04	6.96
**SAN**	**Ohio**	99.23	0.76	96.72	3.27
	**DCLP3**	98.94	1.05	97.33	2.66
	**DCLP5**	97.94	2.058	95.94	4.05
	**RT**	98.12	1.857	93.35	6.64
**CNN**	**Ohio**	98.53	1.47	96.75	3.24
	**DCLP3**	98.48	1.51	97.32	2.67
	**DCLP5**	97.80	2.19	95.94	4.05
	**RT**	97.77	2.23	94.02	5.97
**TCN**	**Ohio**	98.30	1.69	96.43	3.56
	**DCLP3**	98.42	1.57	97.20	2.79
	**DCLP5**	97.79	2.2	95.79	4.20
	**RT**	97.24	2.75	92.23	7.76

### Assessment of generalization ability

Due to the distinctive characteristics and varied demographics, four datasets exhibit differences in BG dynamics. DCLP5 contains only children’s data (6-13), while DCLP3 consists of adolescents (14-19) and adults (20-71 years) data. Ohio includes only adults’ data (20-80 years), whereas all age categories of children, adolescents, and adults’ data are included in RT. The duration of data collection and therapy utilized are distinct. Thus, these variations in data are the primary drivers in this study to evaluate the model’s generalizability. For this, the model trained on one dataset is tested with the other, the result of which is demonstrated in Table 6. Columns in the Table indicate the datasets used to train the model, while the rows indicate the datasets used to evaluate the model for their generalizability. The evaluation results highlight that the RMSE values obtained from the model trained on one dataset and tested on another closely resemble the result obtained when the model was trained and tested exclusively on the dataset used for testing. For instance, LSTM trained with datasets Ohio, DCLP3, DCLP5, and RT separately and assessed each resultant model with Ohio, achieved different RMSE values of 35.19, 35.63, 36.08, 35.12, all values relatively close to the RMSE value 35.19 obtained with the test set of Ohio itself. For more clarity, the visual plot is presented in Fig 11 in right, where CNN and TCN exhibit higher variability in RMSE values across datasets compared to LSTM. In contrast, SAN and FNN consistently show lower and stable RMSE values for each dataset. This consistency suggests that SAN and FNN have the ability to generalize well to new, unseen data. Also, it indicates that these models effectively learn and apply the essential patterns and features of the data during training, making them reliable for making predictions in various scenarios.

**Table 6:** Summary of the predictive performance and statistical test results for models trained with one dataset and tested with the other, denoted by RMSE (p-value). Bold letters indicate the results obtained with the models trained and tested with the same dataset.

		Ohio	DCLP3	DCLP5	RT
**FNN**	**Ohio**	**35.19 (1)**	35.63 (0.53)	36.08 (0.58)	35.12 (0.64)
	**DCLP3**	39.07 (0.54)	**38.81 (1)**	38.87 (0.58)	39.2 (0.6)
	**DCLP5**	47.78 (0.59)	47.15 (0.6)	**47.22 (1)**	47.8 (0.62)
	**RT**	45.58 (0.63)	45.89 (0.55)	46.52 (0.6)	**45.24 (1)**
**LSTM**	**Ohio**	**31.12 (1)**	31.91 (0.53)	32.53 (0.55)	31.23 (0.6)
	**DCLP3**	36.24 (0.55)	**34.91 (1)**	35.01 (0.64)	35.48 (0.62)
	**DCLP5**	45.59 (0.54)	43.99 (0.65)	**43.66 (1)**	44.71 (0.63)
	**RT**	43 (0.6)	42.89 (0.66)	43.3 (0.62)	**41.94 (1)**
**SAN**	**Ohio**	**32.08 (1)**	31.98 (0.55)	32.39 (0.59)	31.91 (0.6)
	**DCLP3**	36.91 (0.54)	**35.23 (1)**	35.41 (0.62)	36.17 (0.6)
	**DCLP5**	46.42 (0.61)	44.55 (0.6)	**44.28 (1)**	45.24 (0.65)
	**RT**	43.6 (0.6)	43.02 (0.63)	43.53 (0.59)	**42.38 (1)**
**CNN**	**Ohio**	**32.4 (1)**	36.21 (0.53)	33.38 (0.62)	32.73 (0.65)
	**DCLP3**	37.19 (0.6)	**38.82 (1)**	36.37 (0.66)	36.97 (0.65)
	**DCLP5**	46.06 (0.61)	46.39 (0.66)	**44.88 (1)**	45.53 (0.68)
	**RT**	43.82 (0.62)	46.94 (0.63)	43.9 (0.63)	**43.18 (1)**
**TCN**	**Ohio**	**32.57 (1)**	35.67 (0.5)	33.79 (0.55)	33.76 (0.62)
	**DCLP3**	36.9 (0.61)	**38.03 (1)**	36.47 (0.64)	37.77 (0.83)
	**DCLP5**	45.58 (0.67)	46.16 (0.65)	**44.97 (1)**	46.08 (0.64)
	**RT**	43.29 (0.55)	46.36 (0.6)	44.26 (0.61)	**44 (1)**

Furthermore, the model trained on one dataset and tested with the other three datasets exhibits similar results as obtained by evaluating it with the test set of the trained dataset showcasing its adaptability and generalization capabilities. The model trained with the dataset DCLP5 appeared to perform best, followed by RT, DCLP3, and Ohio. The models trained with DCLP5 seemed to exhibit adaptability, where its performance is well not only in its original age range (6-13) but also in a broader age range embodied in DCLP3 (14-71 years) and Ohio (20-80 years) including RT that covers entire age range. Even though DCLP5 includes samples of patients of age range beyond what the DCLP3 and Ohio include and is more influenced by the closed-loop control system that offers precise control over BGLs, being able to capture the patterns and variations in data presented in RT that has no precise control over BG suggests a robust generalization capability of the model beyond what was seen during training.

Moreover, since RT includes samples of the entire age range, the performance of the models trained with RT are good across all datasets except DCLP5, where the models generalize reasonably well but are not as effective as others. The models trained with RT achieved higher RMSE with its test set compared to that obtained with the two testing datasets, indicating that the models have learned and captured the underlying trends and patterns in the dataset during training, while slightly higher RMSE for DCLP5 suggests that there might be some differences in the patterns between datasets RT and DCLP5 that RT couldn’t capture. Even though Ohio includes broad diversity in age (20-80), due to its small size, it couldn’t get sufficient exposure to various age groups during training, affecting its performance. Next, models trained with DCLP3 perform significantly well with dataset Ohio, whose age range lies within its original age range; however, the models struggle with datasets DCLP5 and RT and can’t be generalized effectively. The characteristics and distribution of data in DCLP3 may not be able to capture or present the diversity present in DCLP5 and RT, making it challenging for the model to be generalized effectively. Overall, the models trained with DCLP5, and RT generalize more effectively to different datasets than others. However, these models produce higher RMSE values when tested with their own datasets (DCLP5 and RT) compared to testing with other datasets (DCLP3 and Ohio). In addition, models trained and tested with DCLP3, and Ohio have lower RMSE than the models trained and tested with DCLP5 and RT. All these results suggest that the models might be more representative of DCLP3, and Ohio and the relationships present in those datasets, while datasets DCLP5 and RT may have more challenging patterns for the models to interpret. Nevertheless, the models trained with DCLP5, and RT could identify significant features and characteristics that match DCLP3 and Ohio and thus are transferrable between datasets while testing, which is the reason for lower RMSE when tested with DCLP3 and Ohio. From this observation, we believe the models have acquired knowledge to excel in the scenarios beyond what was seen during training. However, for the datasets DCLP5 and RT, due to the higher RMSE result, further analysis of the data distribution and characteristics is required for making decisions on using models and improvements.

To further investigate the generalizability, we examined how well the model performs on different demographics and ensured that models generalize effectively to unseen instances of those subsets. Since dataset RT includes different demographics, we utilized RT for the investigation and investigated the model’s ability to generalize across female and male data. Initially, the models were trained with the training set that has representative distribution of female and male data. The test set that also has a balanced distribution of male and female data is then used to test the models. Further, the models are again evaluated with female and male subsets of the test set separately by using the RMSE performance metric. The result in Table 7 (left), shows the RMSE values obtained with the entire test set, female subset, and male subsets separately, where the male subset has the lowest RMSE while the highest RMSE is observed with female subset, suggesting potential gender bias in the models. To statistically verify the indicated result, statistical analysis for p-values is conducted using the KS test, where p-values for each pair of residuals generated by testing the models with each test set are estimated. Even though the predictive performance shows a significant difference, the p-values exceeding the significance threshold value of 0.05 in Table 7 (right) suggest no statistical difference in the distributions of the residuals between genders, indicating any minimal predictive bias present has an insignificant effect on the prediction errors between genders.

**Table 7:** Generalizability evaluation based on RMSE, and p-values obtained with KS test for female and male demographics in RT dataset. FM, F, and M represent the entire test set, female subset, and male subset, respectively.

	Predictive performance (RMSE)			KS-Test (p-values)		
Model	FM	M	F	FM vs F	FM vs M	F vs M
**FNN**	29.86	27.99	31.92	0.57	0.6	0.51
**LSTM**	26.55	24.63	28.51	0.55	0.59	0.53
**SAN**	27.26	25.37	29.21	0.59	0.63	0.5
**CNN**	29.47	27.57	31.55	0.57	0.59	0.5
**TCN**	34.03	31.87	36.42	0.53	0.57	0.5

### Statistical assessment across datasets

The statistical analysis results for p-values of the KS test for the pair of residuals of models trained on one dataset and tested with another are depicted in Table 6 within square brackets in RMSE (p-value) format. The p-values greater than the significance threshold of 0.05 suggests that the residual distributions of the models trained and tested with the one dataset are not significantly different from the residuals obtained by testing it with other datasets. For instance, the distribution of the residuals of the models trained and tested with Ohio is not statistically significantly different from the distribution of residuals of the models tested with DCLP3, DCLP5, or RT, as demonstrated by the p-values (e.g., 0.54, 0.59, 0.63 for FNN). The results suggest that the models performed statistically consistently across datasets, showcasing their generalizable capabilities.

### Complexity analysis

To analyze the complexity within each model, different time and memory-based complexity, number of parameters (NP), floating point operations (FLOPs), memory footprint (MF), and inference time (IT) is analyzed, which is shown in Table 8. The table indicates that the SAN is computationally expensive and requires significant resources due to its higher number of parameters, extended inference time, large memory footprint, and most FLOPs, limiting its use in resource-constrained environments. The FNN on the other hand is the most efficient in terms of parameter size, memory footprint, and inference time, though its FLOPs are higher compared to LSTM. Both CNN and TCN show moderate complexity across all aspects, with TCN having potentially greater capacity. Overall, the LSTM model seems to have a good balance between efficiency and performance, with the lowest FLOPs.

**Table 8:** Summary of the complexity metrics of the five models.

	NP	FLOPs	MF(MB)	IT(sec)
**FFN**	2241	40464	0.01	0.00167
**LSTM**	11431	1200	0.04	0.002439
**SAN**	595457	4083184	2.27	0.028886
**CNN**	31041	75010	0.12	0.005299
**TCN**	71457	247042	0.27	0.016618

### State-of-the-art comparison

A comparison with different state-of-the-art methods for BGL prediction using the Ohio dataset is depicted in Table 9. Most of the existing works employed Ohio to evaluate the model and considered RMSE as an evaluation metric for PHs of 30 and 60 minutes, thus we presented a comparison based on RMSE for both PHs for the Ohio dataset, where best performing model, LSTM, is considered for the comparison. None of the existing works assessed generalizability across datasets or within datasets, even though some studies [11, 13] utilized more than one dataset to evaluate their model. In terms of performance comparison, the LSTM model in this study outperformed all the existing models for 30-minute PH, except MTL-LSTM. However, for a 60-minute PH, the performance of the LSTM model is slightly higher than FCNN and MTL-LSTM with close resemblance while outperforming all other models.

**Table 9:** Comparison with state-of-the-art BGL predictive methods evaluated with OhioT1DM dataset. RMSE is used for assessment, where bold letters indicate best RMSE.

Models	Input Features	Datasets	Generalizability	RMSE (30-min)	RMSE (60-min)
**Recurrent Self-Attention [10]**	CGM, Carb, Insulin	Ohio	No	18.90	31.52
**FCNN [11]**	CGM, Carb, Insulin	Ohio, ARISES, ABC4D	No	18.64	31.07
**CNN [5]**	CGM,	Ohio, T2DM private	No	19.08	33.80
**CRNN [14]**	CGM, Carb, Insulin,Exercise	Ohio	No	18.8	31.8
**MTL-LSTM [12]**	CGM, Carb, Insulin, Fingerstick BGL	Ohio	No	16.06	30.89
**GRU [49]**	CGM	Ohio	No	21.90	35.10
**Ensemble [15]**	CGM	Ohio	No	19.63	33.45
**E3NN [13]**	CGM,	Ohio, ARISES, ABC4D	No	18.92	32.54
**LSTM (This study)**	CGM	Ohio, DCLP3, DCLP5, RT	Yes	18.26	31.12

## Limitations

Regarding the limitations, this study utilized CGM data as the sole input feature. Thus, in future research, it would be worthwhile to investigate the performance of the models exploring the potential of additional features in an optimized manner. Additionally, investigating datasets to understand various influencing factors, glucose variability, and their impact on prediction would be beneficial. Accurate prediction of adverse events could enhance the management of diabetes with actionable insights. This limitation of this study can be addressed in future by incorporating the ability to predict specific blood glucose events, such as hypoglycemia and hyperglycemia. To further ensure the applicability and usefulness in real-world scenarios, it is essential to consider scalability, interoperability and regularity compliance within the context of clinical relevance.

## Conclusion

This study presented a comprehensive analysis of different deep learning models for generalization capabilities and predictive performance based on diverse datasets for BGL prediction. The results showed that the LSTM and SAN achieved the lowest RMSE with the highest generalization capability among all the models. Despite lower performance in predictive accuracy, FNN captured general patterns and trends in the data and understood the data dynamics. Such models can be applicable where predicting a general direction is more crucial than precise numerical predictions. Also, further refinement such as incorporating additional data features could potentially improve the predictive performance while maintaining the ability to capture underlying patterns. The performance of CNN and TCN in terms of clinical acceptance showed comparable performance with LSTM and SAN, indicating the models’ potential applicability in the BGL prediction task. The assessment of generalization ability including the statistical test indicates that all the models performed statistically consistently across datasets proving their generalizable capability, with consistent performance with SAN followed by LSTM. The superiority in the performance has also been verified by the state-of-the-art performances. Overall, we anticipate that this study establishes a benchmark for BGL predictive tasks and offers researchers an empirical insight into how different models behave and how they can be applied in different patient groups and hospital settings.
